# IL-23 and monocyte interactions redirect human Th17.1 cells to an IL-17A^high^IFN-γ^high^ potentially pathogenic profile

**DOI:** 10.3389/fimmu.2026.1793184

**Published:** 2026-09-30

**Authors:** Bennie van Heeswijk, Wida Razawy, Nadine Davelaar, Anne-Marie M.C. Mus, Patrick S. Asmawidjaja, Pieter J.M. Leenen, Erik Lubberts

**Affiliations:** 1 Department of Rheumatology, Erasmus MC, University Medical Center, Rotterdam, Netherlands; 2 Department of Immunology, Erasmus MC, University Medical Center, Rotterdam, Netherlands

**Keywords:** CCR6, IFN-γ, IL-17A, IL-23, monocyte, plasticity, psoriatic arthritis, Th17.1

## Abstract

**Introduction:**

IL-23–targeted therapies have demonstrated clinical efficacy in psoriatic arthritis (PsA), primarily through the reduction of IL-17A–driven inflammation. Consequently, most studies have focused on classical, high IL-17A-producing, CCR6^+^CXCR3^-^CCR4^+^ Th17 cells. In contrast, the impact of IL‑23 signaling on CCR6^+^CXCR3^+^CCR4^-^ Th17.1 cells —traditionally characterized by high IFN-γ and low IL-17A production—remains largely unexplored, despite their recognized pathogenicity in autoimmune disease. The aim of this study was to characterize the effects of IL-23 on human memory Th17.1 cells.

**Methods:**

Peripheral blood mononuclear cells (PBMCs) from treatment-naive patients with early PsA were stimulated in the presence of IL-23 or anti-IL-23 antibody before quantifying IL-17A and IFN-γ secretion. Additionally, human memory CCR6^-^ Th1, CCR6^+^ Th17.1 and CCR6+ Th17 cells from healthy donors were sorted and analyzed ex vivo for *IL23R*, *TBX21* and *RORC* expression using RT-qPCR. Sorted Th17.1, Th17 and Th1 cells were subsequently cultured alone or cocultured with IL-23-producing monocytes. Cultures received either exogenous IL-23 or IL-23 inhibition before analyzing IL-17A and IFN-γ secretion.

**Results:**

As expected, IL-23 stimulation induced IL-17A in PsA PBMCs, whereas IL-23 inhibition reduced IL-17A secretion but did not affect IFN-γ. In healthy donor PBMCs, ex vivo IL-23R expression was restricted to memory CCR6^+^ Th17 and Th17.1 cells. Remarkably, IL-23 stimulation of Th17.1 cells significantly increased IFN-γ without affecting IL-17A secretion. Compared to monoculture, cocultures with monocytes increased IFN-γ secretion in Th17.1 and classical Th1 cells, and IL-17A secretion in Th17 and Th17.1 cells. Additional IL-23 stimulated IL-17A in both Th17 and Th17.1 cocultures, while unexpectedly reducing IFN-γ in Th17.1 cells. In contrast, IL-23 inhibition reduced IL-17A secretion in Th17 and Th17.1 cells without affecting IFN-γ production.

**Conclusion:**

IL-23 exerts distinct effects on Th17.1 compared to Th17 cells. We show a role for IL-23 in IFN-γ upregulation in Th17.1 cells, which is reversed in the presence of monocytes. In co-culture with monocytes, Th17.1 cells progress to a pathogenic IL-17A^high^IFN-γ^high^ profile, which is further elevated by IL-23 stimulation and reduced upon IL-23 neutralization. We show that IL-23 drives Th17.1 plasticity and underscore the therapeutic relevance of neutralizing IL-23 in the suppression of these pathogenic IL-17A^high^IFN-γ^high^ Th17.1 cells.

## 1 Introduction

The IL-23/Th17 axis is a well-established driver of chronic inflammation (1–3). IL-23 activates STAT3-dependent programs through IL-12Rβ1 and IL-23R and promotes Th17 cell survival, proliferation, and effector function. Blockade of IL-23 has demonstrated significant clinical benefit in psoriatic arthritis (PsA) and related disorders, underscoring the pathogenic relevance of this pathway (4). These effects are largely attributed to the suppression of interleukin-17A (IL-17A)-driven pathology (5). Therefore, most studies have focused on the high IL-17A-producing classical Th17 cells, leaving the responsiveness of Th17.1 cells to IL-23 largely uncharacterized (6).

CD4^+^ memory T helper (Th) cells are central coordinators of adaptive immunity, and their functional diversity underlies both protective and pathogenic immune responses. The Th17 lineage, initially described as a heterogeneous population of human IL-17–producing T cells (7, 8) and later further delineated into distinct subpopulations (9), is defined by the expression of surface CCR6 and the master regulator RORγt, has emerged as a critical driver of chronic inflammation and autoimmunity (10). Memory CCR6^+^ CD4^+^ T cells are include both classical CCR6^+^CXCR3^-^CCR4^+^ Th17 cells and CCR6^+^CXCR3^+^CCR4^-^ Th17.1 cells (1). The Th17.1 cell population co-expresses RORγt and the Th1-associated transcription factor T-bet, resulting in high interferon-γ (IFN-γ) but low IL-17A secretion (11). These cells are considered highly pathogenic in autoimmune diseases due to their inflammatory functionality (12), resistance to steroids (13), and capacity to infiltrate inflamed tissues (14). Therefore, this warrants the need to understand the direct effect of IL-23 on Th17.1 cell profile characteristics (6).

IL-23 can be produced by monocytes (15), which are abundant in PsA synovial fluid (16). Additionally, monocytes produce other pro-inflammatory mediators and provide costimulatory signals that further enhance T cell activation (17). Often, the effect of IL-23 on T cells is studied in isolation, but monocyte interaction may influence the effect of IL-23 signaling (18). Monocyte-derived IL-23 is likely to act in concert with additional cytokines such as TNF (19) and IL-6 (20), as well as cell–cell contact–dependent cues, thereby shaping the magnitude of Th17 and Th17.1 cell responses. Understanding how monocyte–T cell interactions and physiologically relevant levels of IL-23 influence the Th17.1 profile is pivotal for understanding IL-23–driven pathology.

In this study, we address this gap by investigating IL-23 responsiveness in human memory CD4^+^ T cells, focusing on Th17, Th17.1, and Th1 cells. We confirm the stimulatory role of IL-23 signaling on IL-17A secretion in PBMC from treatment-naive early PsA patients. Next, we assess IL-23R expression, measure functional responses to IL-23 in T cell cultures and in co-cultures with monocytes, and evaluate the effects of anti-IL-23 treatment. Together, our results provide new insights into IL-23-programmed Th17.1 cell profile expression and functionality and highlight the relevance of Th17.1 cells in IL-23–driven inflammation.

## 2 Material and methods

### 2.1 Subjects

Peripheral blood mononuclear cells (PBMC) from healthy individuals were isolated from buffy coats (Sanquin Bloodbank, Rotterdam, the Netherlands). Eight PsA PBMC were isolated from patients with a new diagnosis of PsA (≥ 18 years) who had not received systemic treatment with disease-modifying anti-rheumatic drugs (DMARDs) or biologicals for joint symptoms earlier. Patient characteristics are shown in **Supplementary Table 1**. Written informed consent was obtained from all participants in accordance with the Declaration of Helsinki.

### 2.2 Cell sorting

Before sorting, CD4^+^ cells were pre-purified from PBMC using CD4 microbeads and magnetic separation (autoMACS, Miltenyi Biotec) following manufacturer’s instructions. Fluorochrome-labeled monoclonal antibodies: CD3-BV785, CD4-FITC, CCR4-APC, CD25-PE-Cy7 (Sony), CD127-PE-Cy5, CD45RO-Bv711 (BioLegend, San Diego, CA, USA), CXCR3-PE-Efluor610 (BD Biosciences), CCR6-PE (Thermo Fisher Scientific) were used to sort cells. Dead cells were excluded using 4′6-diamidino-2-phenylindole dilactate (DAPI). For sorting of memory T helper cells, CD3^+^CD4^+^CD45RO^+^CD25^low/int^CD127^+^ cells were selected and further sorted into CCR6^+^, CCR6^-^, CCR6^+^CCR4^+^CXCR3^-^ (Th17), CCR6^+^CCR4^-^CXCR3^+^ (Th17.1) or CCR6^-^CCR4^-^CXCR3^+^ (Th1) cells. Cells were sorted using a FACS Aria III flow cytometer (BD Biosciences) using the sorting strategy shown in **Supplementary Figure 1**. CD14^+^ monocytes were isolated from PBMC using CD14 microbeads (autoMACS, Miltenyi Biotec) following manufacturer’s instructions, and purity was verified (>95%) using CD14 staining and flow cytometry.

### 2.3 Cell culture

Sorted memory T helper cells were cultured at a density of 25.000 or 50.000 cells/well in round-bottom 96-well plates. For co-culture with monocytes, T cells and monocytes (25.000 each per well) were cultured 1:1 in flat-bottom 96-well plates. Cells were cultured for three days in Iscove’s Modified Dulbecco’s Medium (IMDM) supplemented with 10% fetal calf serum, 2 mM L-glutamine (Lonza), 100 U/ml penicillin-streptomycin and 50 μM β-mercaptoethanol (Sigma Aldrich, USA). Sorted cells were stimulated with soluble αCD3 (0.3 μg/ml; Sanquin or 0.4µg/ml; Miltenyi Biotech) and CD28 antibodies (0.4 μg/ml; Sanquin or 0.6µg/ml; Miltenyi Biotech) with or without 30 ng/ml recombinant IL-23 (R&D systems, USA) or 0.1 µg/ml anti-IL-23 (Guselkumab, Thermo Fisher Scientific). Data points in all graphs respond to individual donors.

### 2.4 ELISA

The concentration of IL-17A, IL-22, IL-10, TNFα, GM-CSF (Thermo Fisher Scientific) and IFN-γ (Thermo Fisher Scientific and R&D systems, USA) was measured in culture supernatant by ELISA according to manufacturer’s instructions.

### 2.5 Real-time PCR

RNA was extracted using the GenElute Mammalian Total RNA Miniprep Kit (Sigma Aldrich) according to manufacturer’s instructions. Isolated RNA was treated with 0.1 U/µl DNaseI (Invitrogen). Subsequently, the RNA was reverse transcribed into cDNA using random hexamer primers and Superscript II (Invitrogen). Gene-specific primers were designed with ProbeFinder software (Roche Applied Science, USA) and probes were used from the Universal Probe Library (Roche Applied Science, USA). HPRT was used to normalize gene expression. Primers and probes that were used are listed in **Table 1**. Real-time qPCR was performed using the Viia7 system (Applied Biosystems, USA) and data were analyzed using QuantStudio Real-time PCR software version 1.3 (Applied Biosystems, USA).

**Table 1:** Used primer sequences and probes.

Gene	Primer 1	Primer 2	Probe #
*HPRT*	TGACCTTGATTTATTTTGCATACC	CGAGCAAGACGTTCAGTCCT	73
*IL23R*	TGAAGTGGAATTATGTGCTTCAA	GCATCCCATTGAATAGTGACC	38
*IL12RB1*	AGTTCCTGGAGCAAGTGGAG	AGCTGCTCCACCGAGAATC	90
*IL12RB2*	GACTCAGATATCGGCCCAGT	TGTCTTCCTTTGGCCTTTGTA	40
*RORC*	CAGCGCTCCAACATCTTCT	CCACATCTCCCACATGGACT	69
*TBX21*	TGTGGTCCAAGTTTAATCAGCA	TGACAGGAATGGGAACATCC	9

### 2.6 Statistical analysis

Differences between experimental groups were tested with a two-sided paired Student t-test or as stated otherwise, using R (R version 4.4.3). Data are depicted as mean ± SEM and p-values< 0.05 are considered statistically significant.

## 3 Results

### 3.1 IL-23 modulates IL-17A secretion in PBMCs from treatment-naive psoriatic arthritis patients

To investigate the role of IL-23 in psoriatic arthritis (PsA), PBMC from treatment-naive patients with early PsA were stimulated with αCD3 and αCD28 antibodies with or without IL-23, isotype control or a specific neutralizing IL-23 Ab. As expected, IL-23 stimulation led to a marked increase in IL-17A secretion compared to controls (**Figure 1A**), demonstrating that circulating immune cells in PsA are responsive to IL-23 and capable of initiating a type-17 response. In contrast, IFN-γ secretion remained unchanged (**Figure 1B**). Neutralizing IL-23 did not affect IFN-γ levels (**Figure 1D**) but significantly reduced IL-17A secretion compared to isotype control (**Figure 1C**), confirming the contribution of endogenous IL-23 to IL-17A production in these mononuclear cells of PsA patients.

**Figure 1:**
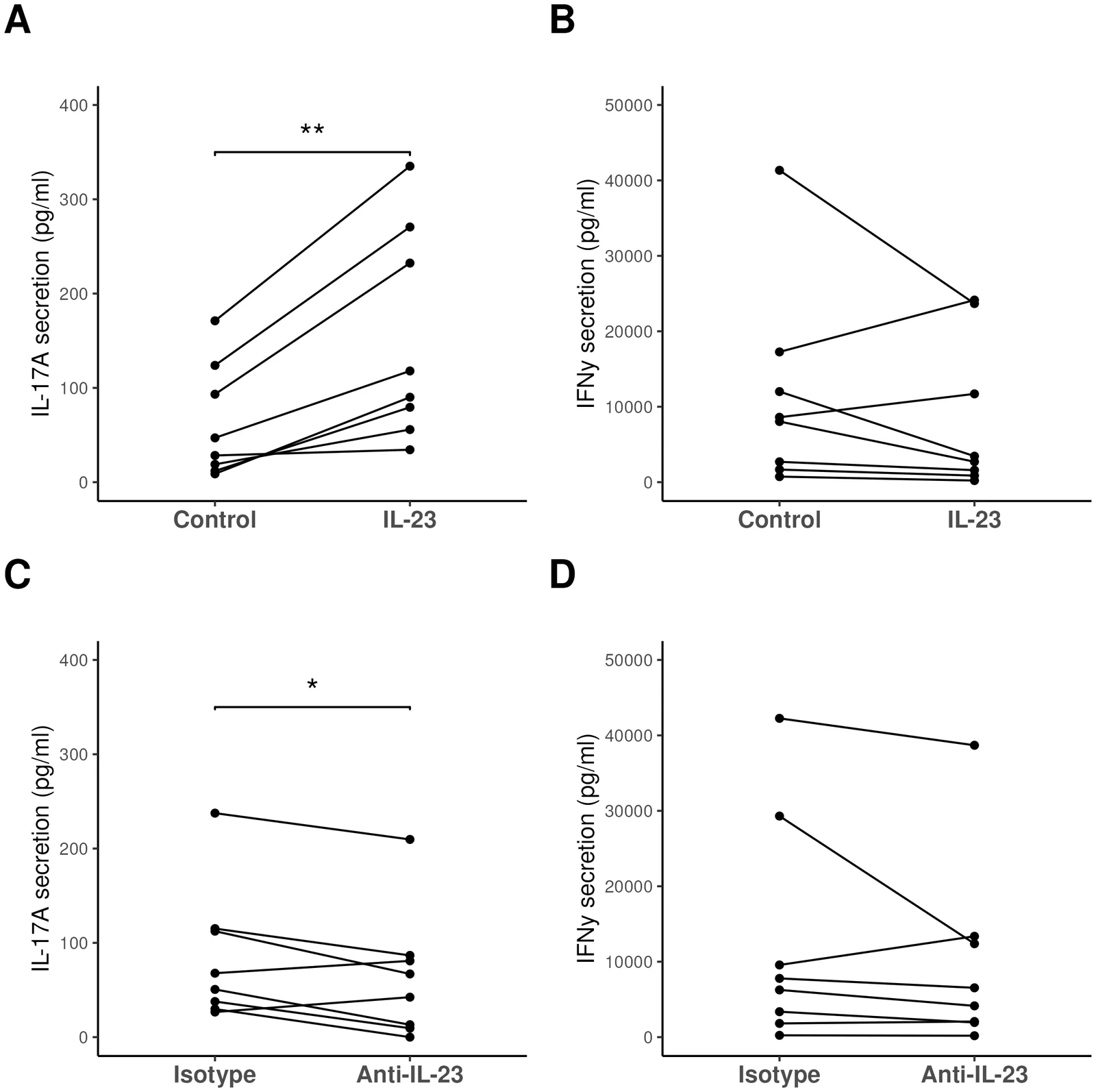
PBMCs from treatment-naive patients with psoriatic arthritis highlight the association between IL-23 and IL-17A. PBMCs were stimulated with αCD3/αCD28 antibodies in the presence or absence of IL-23 for 72 hours. **(A)** IL-17A and **(B)** IFN-γ levels were measured using ELISA. **(B)** PBMCs were stimulated with αCD3/αCD28 antibodies and treated with a specific anti-IL-23 Ab or isotype control before measuring **(C)** IL-17A and **(D)** IFN-γ. Data represents two independent experiments, with a total sample size of n = 7–8. Paired t-tests were used for comparing cytokine levels between two groups. * P<0.05 and ** P<0.01.

### 3.2 Human CCR6^+^ memory T cells exhibit elevated IL23R expression and Th17 identity

To identify Th cell subsets responsive to IL-23, we analyzed the expression of IL-12/IL-23 receptor subunits—*IL12RB1*, *IL12RB2*, and *IL23R*—in sorted human memory CCR6^+^ and CCR6^–^ CD4^+^ T cells from healthy donors (**Figure 2**). Memory CCR6^+^ T helper cells showed significantly higher expression of *IL23R* and *IL12RB2* compared to their CCR6^–^ counterparts, while *IL12RB1* was similarly expressed across both populations. This suggests the presence of both IL-12 and IL-23 receptor complexes (IL-12Rβ1/β2 and IL-23R/IL-12Rβ1, resp.) in the memory CCR6^+^ Th cells, while CCR6- Th cells only express the IL12 receptor complex. To characterize the functional identity of these cells further, we assessed transcription factors associated with T helper cell lineages. RORC, which encodes RORγt, the master regulator of Th17 cells, was elevated in memory CCR6^+^ Th cells, reinforcing their Th17-like phenotype. T-bet, defined by the TBX21 gene and associated with Th1 and Th17.1 cells, was expressed in both memory CCR6^+^ and CCR6^–^ Th cells, indicating some degree of lineage overlap or plasticity.

**Figure 2:**
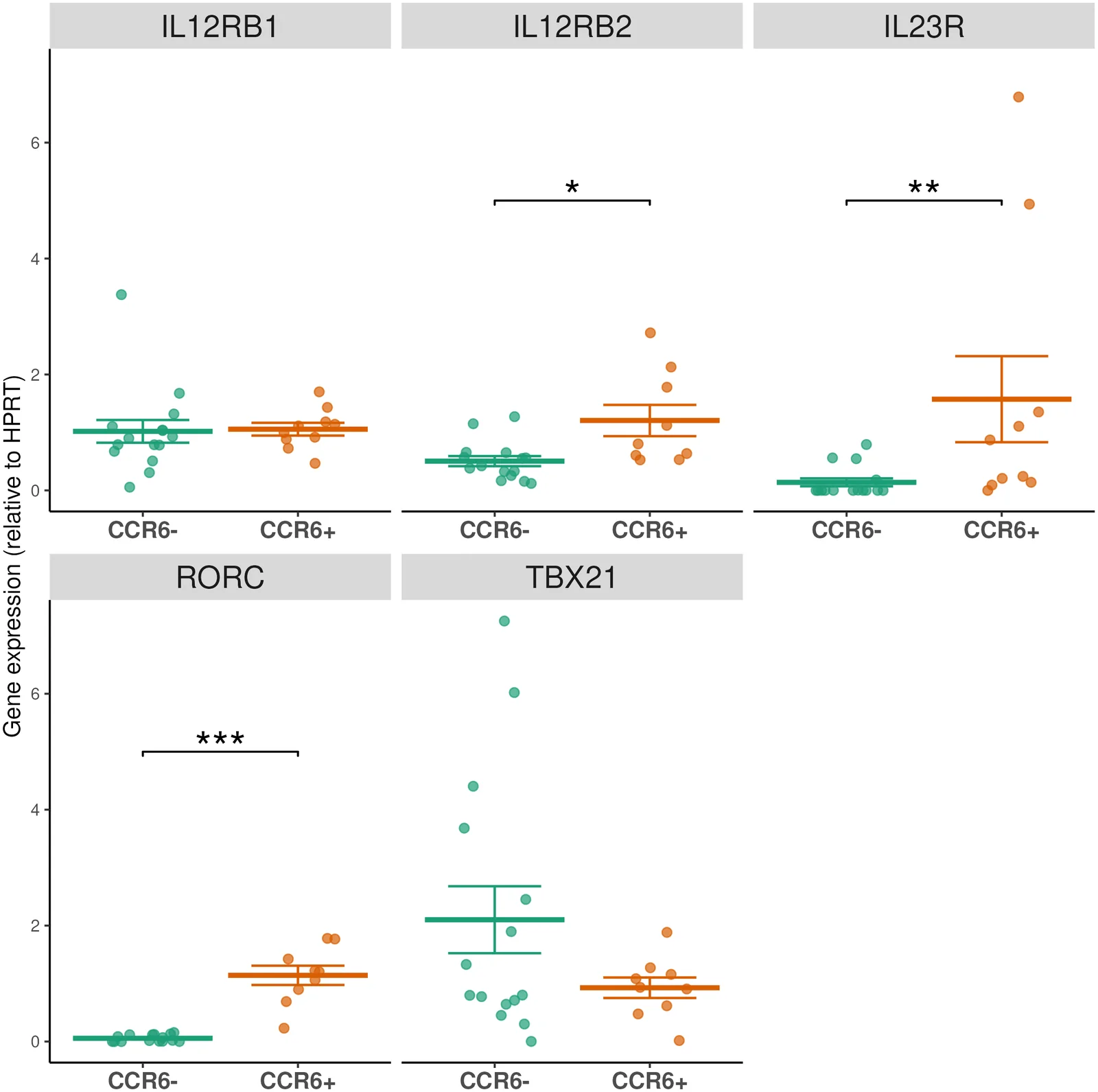
Expression of IL-23 receptor subunits and transcription factors in human memory CCR6^+^ and CCR6^–^ CD4^+^ T cells. Sorted CCR6^+^ and CCR6^–^ memory CD4^+^ T cells from healthy donors were analyzed for *IL23R*, *IL12RB1*, *IL12RB2*, *RORC* and *TBX21* expression by qPCR. Data represents three independent experiments, with a total sample size of n = 9–15. Data are presented as mean ± SEM. Statistical analyses were performed using Wilcoxon signed-rank tests. * P<0.05, ** P<0.01, *** P<0.001.

### 3.3 IL-23 stimulation enhances IFN-γ secretion in CCR6^+^ memory Th cells

To assess the functional relevance of IL-23 responsiveness in memory CCR6^+^ Th cells, we next examined cytokine production following *in vitro* stimulation. Human memory CCR6^+^ and CCR6^–^ Th cells were activated with αCD3 and αCD28 antibodies, in the presence or absence of IL-23 for 72 hours. Surprisingly, memory CCR6^+^ Th cells showed a significant increase in IFN-γ secretion upon IL-23 stimulation, whereas CCR6^–^ cells remained unaffected (**Figure 3B**). IL-23 stimulation did not significantly alter the secretion of IL-17A, IL-22, TNFα, or IL-10 in either Th cell group (**Figures 3A, C, D, E**). This finding challenges the conventional view that IL-23 primarily drives IL-17A production in humans, as it demonstrates that IL-23 can enhance Th1-like effector functions by promoting IFN-γ secretion in human memory CCR6^+^ Th cells.

**Figure 3:**
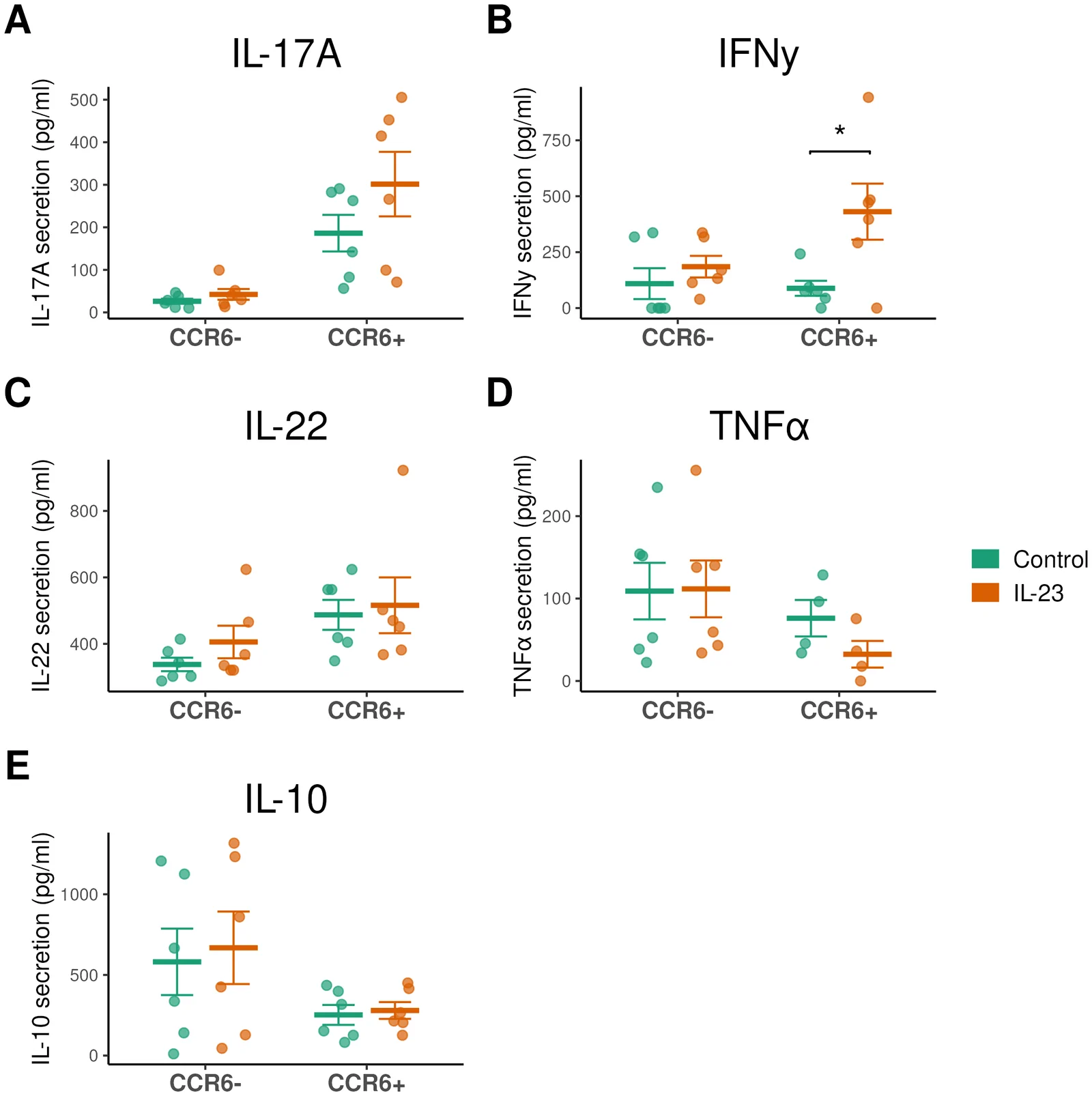
IL-23 stimulates IFN-γ secretion in sorted CCR6+ memory Th cells. Sorted human memory CCR6^+^ and CCR6^–^ Th cells were stimulated with αCD3/αCD28 antibodies for 72 h in the presence or absence of IL-23 before measuring cytokine levels of **(A)** IL-17A, **(B)** IFN-γ, **(C)** IL-22, **(D)** TNFα and **(E)** IL-10 using specific ELISAs. Data are presented as mean ± SEM. Data represent two independent experiments, with a total sample size of n = 4-6. Paired t-tests were used for comparing cytokine levels between two groups. * P<0.05.

### 3.4 IL-23 receptor is expressed in both Th17 and Th17.1 subsets

Given the transcriptional and functional heterogeneity within the human memory CCR6^+^ Th cell population, we further sorted this compartment into Th17 and Th17.1 subpopulations and compared them to classical IFN-γ-producing Th1 cells to understand IL-23 receptor complex expression better. *IL12RB1* was similarly expressed across all groups, indicating general availability. *IL12RB2* was more highly expressed in Th1 and Th17.1 cells compared to Th17 cells, while *IL23R* was expressed in both Th17 and Th17.1 cells, but not in Th1 cells (**Figure 4**). Although not statistically significant, a trend emerged suggesting that Th17.1 cells express more *IL23R* than Th17 cells, suggesting a higher capacity for IL-23 responsiveness. *RORC* was highly expressed in Th17 and Th17.1 cells, whereas *TBX21* expression was higher in Th1 and Th17.1 cells, aligning with IFN-γ secretion by these populations.

**Figure 4:**
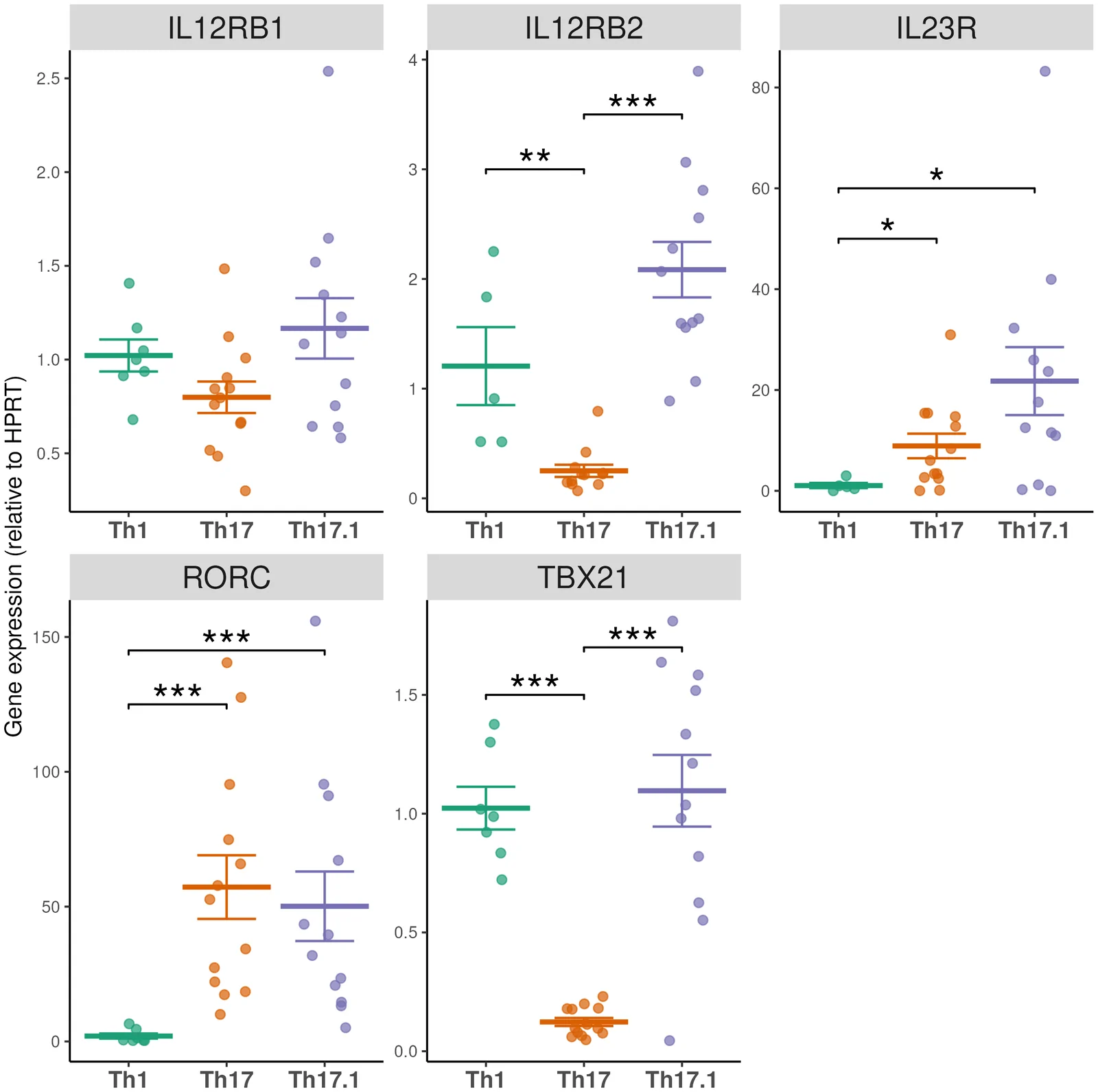
Expression of IL-23 receptor subunit and transcription factor genes in Th17, Th17.1, and Th1 subsets. Sorted human memory Th17, Th17.1, and Th1 cells were analyzed for *IL12RB1*, *IL12RB2*, *IL23R*, *RORC* and *TBX21* expression by qPCR. Data represents three independent experiments, with a total sample size of n = 5–13. Data are presented as mean ± SEM. Wilcoxon signed-rank tests were used for comparing cytokine levels between two groups.

### 3.5 IL-23 induces IFN-γ secretion in Th17.1 cells, but not in classical Th1 cells

To determine whether IL-23 differentially modulates effector functions across T helper subpopulations, we assessed IFN-γ and IL-17A secretion in sorted human memory Th17, Th17.1, and Th1 cells following exogenous IL-23 stimulation. As expected, classical Th17 cells did not produce IFN-γ, and Th1 cells did not produce IL-17A, while Th17.1 cells secreted both cytokines, confirming a unique profile (**Figure 5**). Upon IL-23 stimulation, only Th17.1 cells exhibited a significant increase in IFN-γ secretion, indicating that IL-23 specifically can enhance Th1-like effector functions in this subpopulation. In contrast, IL-17A secretion remained unchanged in both Th17 and Th17.1 cells, suggesting that IL-23 alone is insufficient to amplify IL-17A further in this context. Consistent with these findings, intracellular cytokine staining (**Supplementary Figure 2**) showed that the frequency of IFN-γ^+^ Th17.1 cells remained similar upon IL-23 stimulation, whereas the mean fluorescence intensity (MFI) of IFN-γ increased. In contrast, both the frequency and MFI of IL-17A^+^ cells were unchanged.

**Figure 5:**
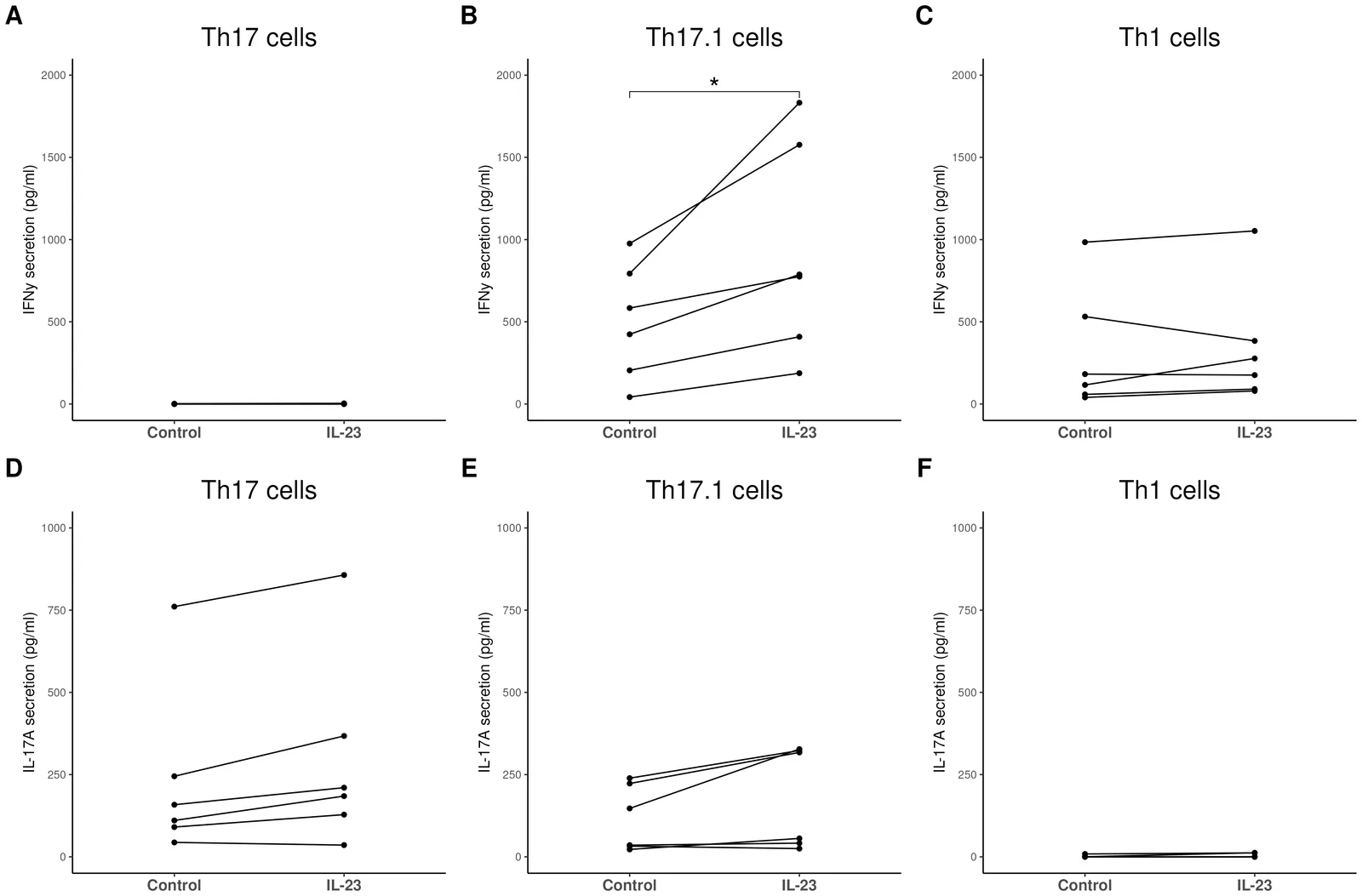
IFN-γ and IL-17A secretion by sorted Th17, Th17.1, and Th1 cells following IL-23 stimulation. Sorted human memory Th17, Th17.1, and Th1 cells were stimulated with αCD3/αCD28 antibodies in the presence or absence of IL-23. **(A, B, C)** IFN-γ and **(D, E, F)** IL-17A levels were measured in culture supernatants after 72 hours. Data represents three independent experiments, with a total sample size of n = 6. Paired t-tests were used for comparing cytokine levels between two groups **(B–E)**; if the data were not normally distributed, the Wilcoxon signed-rank test was applied **(A, F)**. * P<0.05.

### 3.6 Monocytes enhance Th cell effector function in a subset-specific manner

Monocytes are key players in shaping T cell responses through cytokines like IL-23 and costimulatory signals. To examine their impact, we co-cultured sorted human Th17, Th17.1, and Th1 cells with autologous monocytes and measured IL-17A and IFN-γ. Monocytes cultured alone did not produce detectable IL‑17A or IFN‑γ (**Supplementary Figure 3**) The Th17 and Th17.1 cells significantly increased IL-17A secretion upon coculture with monocytes (**Figure 6A**), while IFN-γ was significantly increased in Th1 and Th17.1 cells (**Figure 6B**). Notably, Th17.1 cells secreted significantly more IFN-γ than Th1 cells upon coculturing with monocytes, indicating the strong inflammatory potential of Th17.1 cells.

**Figure 6:**
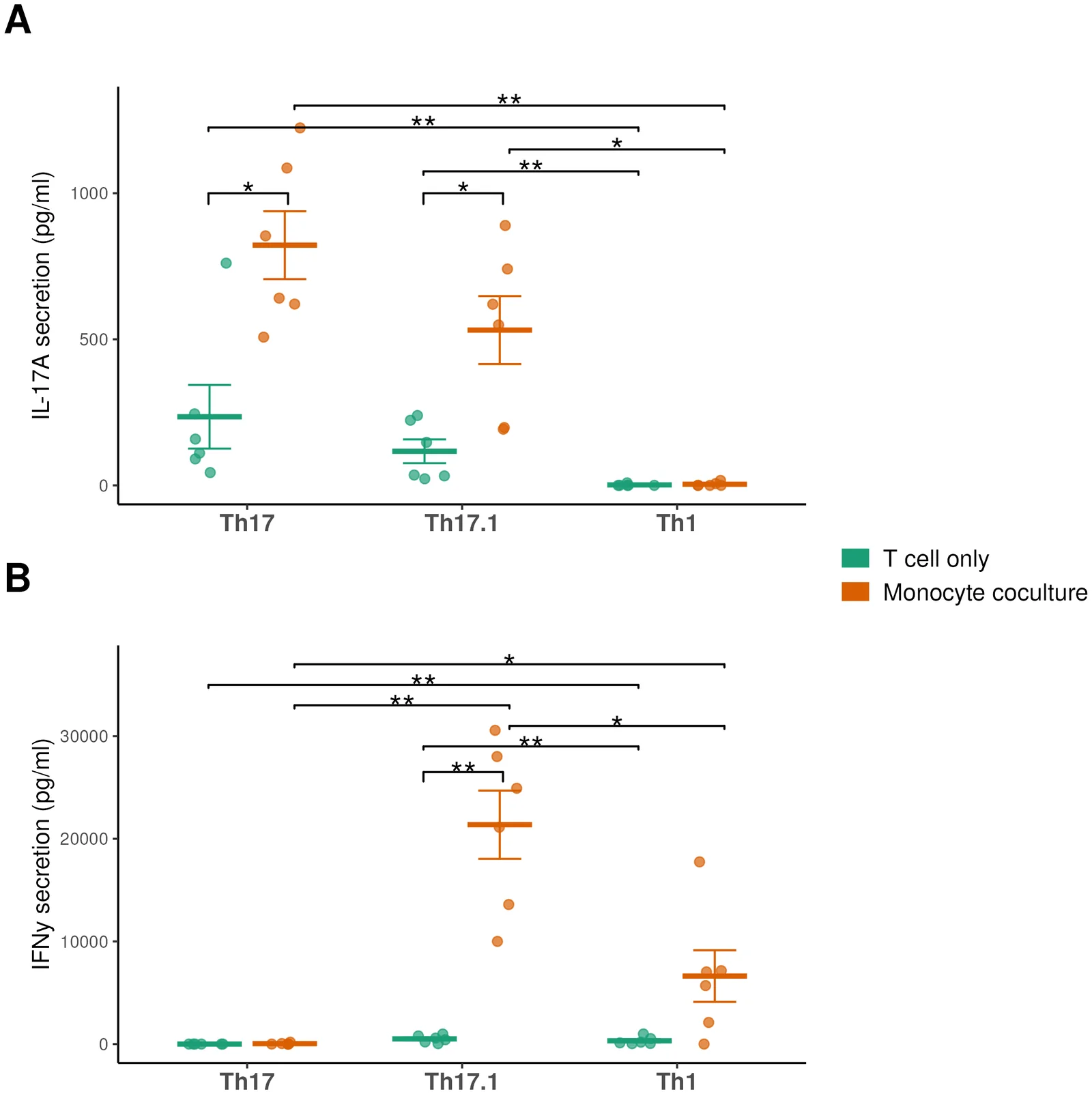
IL-17A and IFN-γ secretion increases upon coculturing T helper subsets with monocytes. Sorted human memory Th17, Th17.1, and Th1 cells were co-cultured with autologous monocytes and stimulated with αCD3/αCD28 antibodies for 72 hours. **(A)** IL-17A and **(B)** IFN-γ levels were measured in culture supernatants. Data represents three independent experiments, with a total sample size of n = 6-8. Data are presented as mean ± SEM. Paired t-tests were used to compare cytokine levels between two groups; if the data were not normally distributed, the Wilcoxon signed-rank test was applied **(A)** Comparisons including Th1 cells, and **(B)** Comparisons including Th17 cells). * P<0.05 and ** P<0.01.

### 3.7 IL-23 shifts Th17.1 cytokine output toward IL-17A while reducing IFN-γ in monocyte co-cultures

Because IL-23 levels can be elevated in autoimmune conditions such as PsA, we introduced exogenous IL-23 into our monocyte–T cell co-cultures to mimic a high IL-23 environment and uncover how strong IL-23 signaling influences T helper cell effector programs. We stimulated sorted human memory Th17, Th17.1, and Th1 cells with or without IL-23 in a monocyte-coculture and measured IL-17A and IFN-γ secretion. IL-23 significantly increased IL-17A secretion in co-cultures with Th17 or Th17.1 cells (**Figures 7D–F**). Unexpectedly, IFN-γ secretion by Th17.1 cells was reduced upon IL-23 stimulation in the monocyte-coculture (**Figures 7A–C**), whereas an increase was observed in Th17.1-only cultures (**Figure 5B**). This suggests that high IL-23 levels, in the presence of monocytes, suppress Th1-like functions in Th17.1 cells and redirects their effector program toward a more Th17-like phenotype, as indicated by the significantly lower IFN-γ/IL-17A ratio upon IL-23 stimulation (**Figure 7G**). In line with this, intracellular cytokine staining of Th17.1 cells showed a reduction in the frequency of IFN-γ^+^ cells and a modest increase in IL-17A^+^ cells upon IL-23 stimulation, while IFN-γ MFI remained comparable and IL-17A MFI showed an increasing trend (**Supplementary Figure 4**). To better understand whether monocyte-derived signals could contribute to these context-dependent effects, we assessed cytokines known to influence Th1 and Th17 responses within the co-culture. While IL-12, a key driver of IFN-γ production, was not detected, both IL-6 and IL-1β were present in the cultures (**Supplementary Figure 5**). These findings highlight the context-dependent effect of IL-23 on Th17 and Th17.1 cells, which is partly determined by signals originating from monocytes.

**Figure 7:**
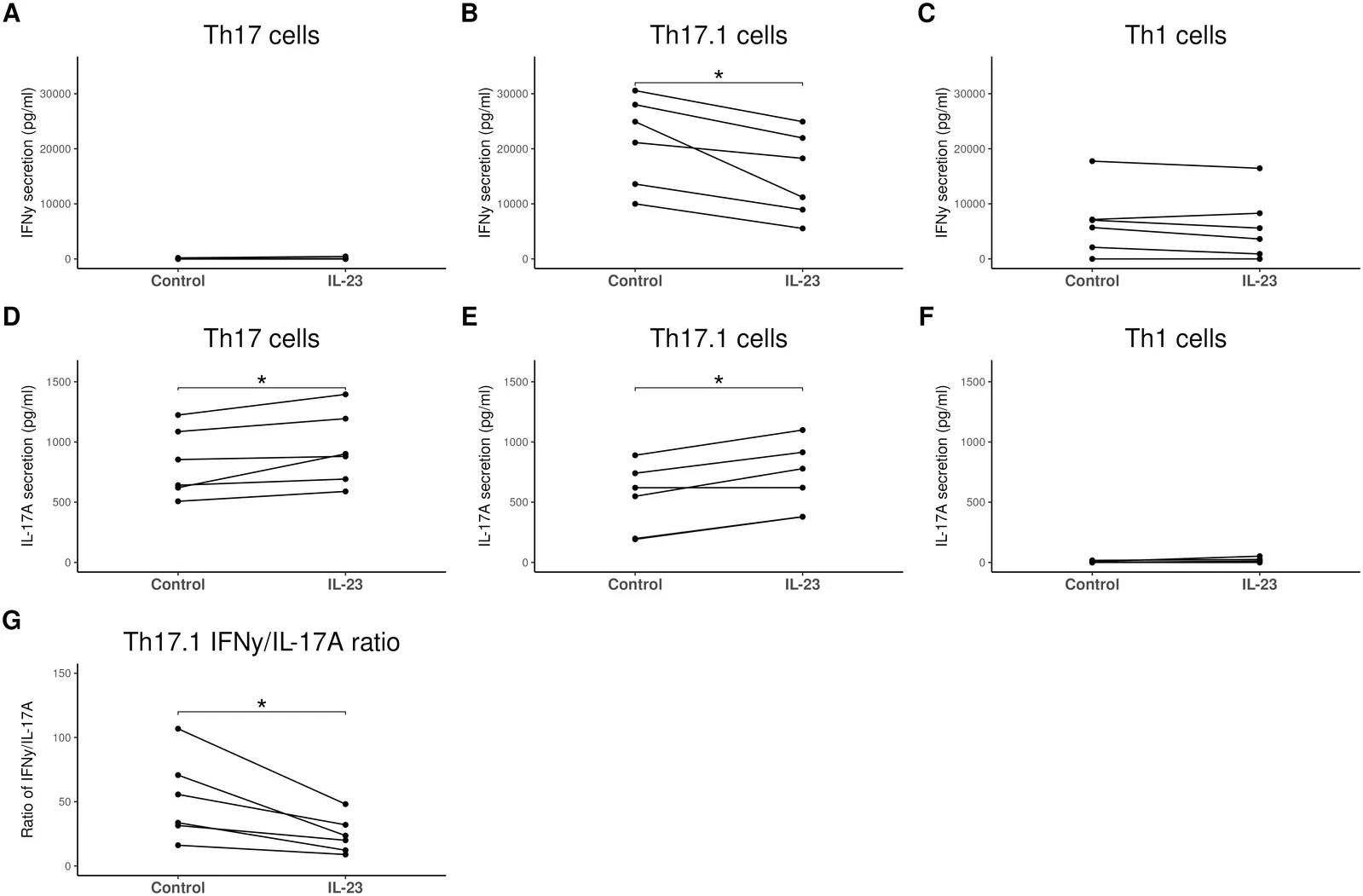
IL-23 stimulates IL-17A but downregulates IFN-γ secretion by Th17.1 cells in co-cultures with monocytes. Sorted human memory Th17, Th17.1, and Th1 cells were co-cultured with monocytes and stimulated with or without IL-23 for 72 hours. **(A, B, C)** IFN-γ and **(D, E, F)** IL-17A levels were measured in culture supernatants. **(G)** IFN-γ/IL-17A level ratio was calculated for Th17.1 cells. Data represents two independent experiments, with a total sample size of n = 6. Paired t-tests were used for comparing cytokine levels between two groups **(B-E, G)** if the data were not normally distributed, the Wilcoxon signed-rank test was applied **(A, F)**. * P<0.05.

### 3.8 Neutralizing monocyte-derived IL-23 reduces IL-17A secretion in Th17 and Th17.1 cells

To assess the effect of endogenous levels of monocyte-derived IL-23, we co-cultured monocytes with sorted human memory Th17, Th17.1, and Th1 cells in the presence of a specific IL-23-neutralizing Ab or an isotype control and measured IL-17A and IFN-γ secretion. IL-17A secretion was significantly reduced in both Th17 and Th17.1 cells upon IL-23 blockade (**Figures 8D, E**), confirming that physiologic levels of monocyte-derived IL-23 support IL-17A production in these cells. In contrast, IFN-γ secretion was not significantly affected by IL-23 blockade in any of the co-cultures (**Figures 8A–C**). This was particularly notable for Th17.1 cells, where additional IL-23 enhanced IFN-γ in monoculture but decreased it in co-culture (**Figure 5B** vs. **Figure 7B**). For Th17.1 cells, the ratio of IFN-γ/IL-17A is increased upon IL-23 neutralization (**Figure 8G**), suggesting a shift toward a more Th1-like functional profile as IL-17A production declines while IFN-γ remains relatively unaffected. Together, these findings demonstrate that IL-23 produced by monocytes is functionally relevant for enhancing IL-17A secretion in human Th17 and Th17.1 cells but has limited influence on IFN-γ, underscoring that IL-23 selectively amplifies Th17-driven responses in environments where monocytes are present.

**Figure 8:**
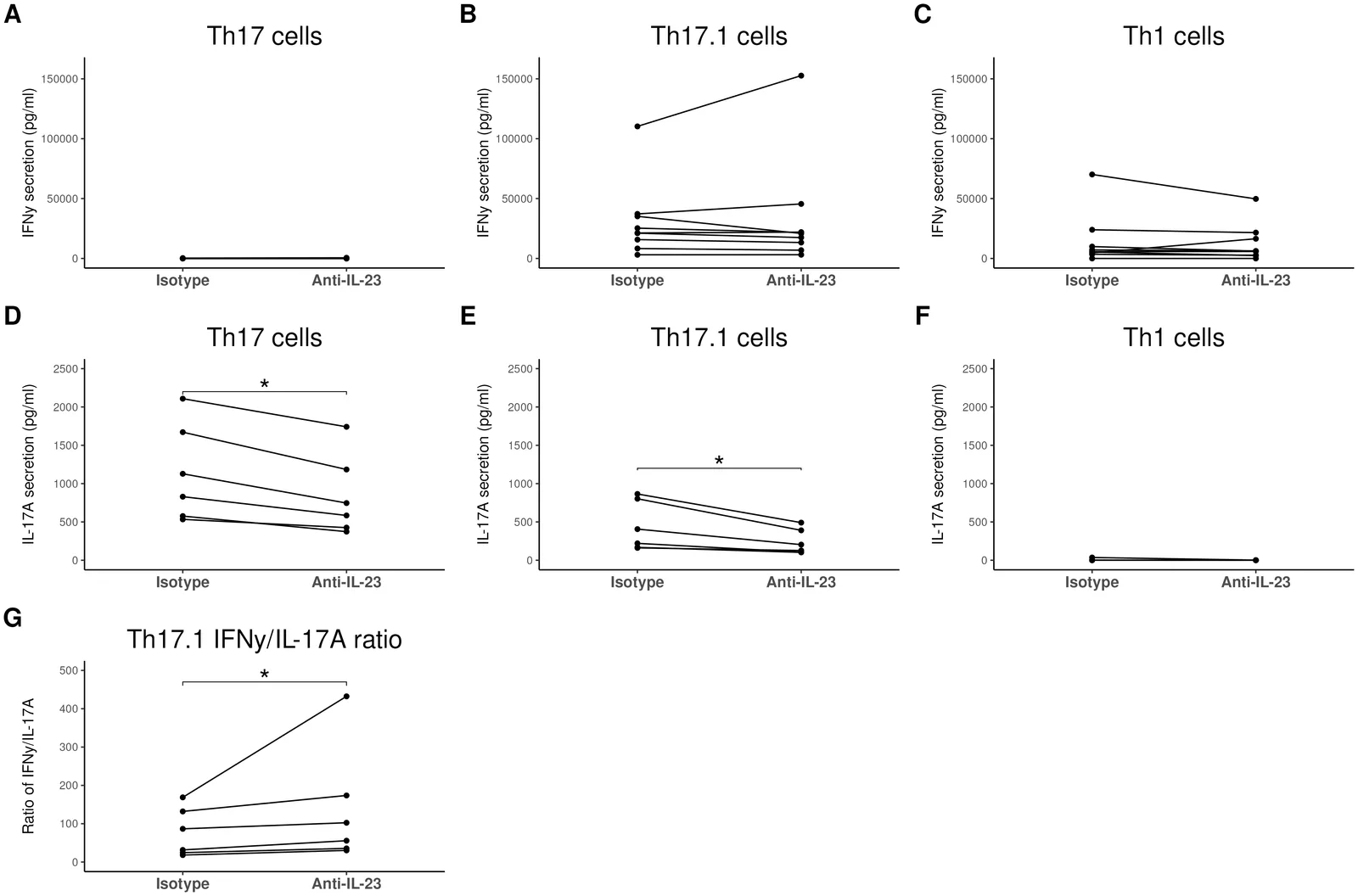
Neutralizing IL-23 downregulates IL-17A in Th17.1 and Th17 cells in co-cultures with monocytes. Sorted human memory Th17, Th17.1, and Th1 cells were co-cultured with monocytes in the presence of a specific neutralizing IL-23 Ab or isotype control for 72 hours. **(A, B, C)** IFN-γ and **(D, E, F)** IL-17A levels were measured in culture supernatants. **(G)** IFN-γ/IL-17A level ratio was calculated for Th17.1 cells. Data represents three independent experiments, with a total sample size of n = 9. Paired t-tests were used for comparing cytokine levels between two groups **(B-E, G)** if the data were not normally distributed, the Wilcoxon signed-rank test was applied **(A, F)**. * P<0.05.

## 4 Discussion

This study uncovers a previously underappreciated dimension of IL-23 biology, showing that its effects on human memory Th17 subpopulations are highly context-dependent. While IL-23 is widely recognized as a driver of Th17-mediated inflammation in psoriatic arthritis (PsA), our findings show that its effects extend beyond classical IL-17A promotion. Notably, we show that IL-23 directly induces IFN-γ production in Th17.1 cells in monoculture, revealing an additional IL-23–responsive effector function in this population. Furthermore, our findings demonstrate that IL-23 signaling, in addition to monocyte-derived cues, dynamically modulates Th17.1 cell plasticity by transforming human IL-17A^low^IFN-γ^high^ Th17.1 cells into an IL-17A^high^IFN-γ^high^ phenotype, highlighting a previously unrecognized pathway of pathogenic reprogramming with direct implications for IL-23 blockade in psoriatic arthritis and potentially other immune-mediated inflammatory diseases.

The clinical relevance of IL-23 signaling is emphasized by the success of IL-23 inhibitors in PsA and other immune-mediated inflammatory diseases (4). Guselkumab and other IL-23p19-targeting agents have demonstrated robust efficacy in reducing IL-17A-driven pathology (5). Our data provide mechanistic support for these observations by showing that IL-23 blockade significantly reduces IL-17A secretion in PBMCs from treatment-naive PsA patients, whereas additional IL-23 stimulation increases IL-17A secretion by these cells.

IL-23R was not expressed by human Th1 cells in contrast to memory CCR6^+^ Th cells, including both Th17 and Th17.1 cells, indicating the potential of the latter cells to mediate IL-23 signaling. Although the difference was not significant, Th17.1 cells tended to express higher levels of IL-23R than Th17 cells, suggesting a greater potential for IL-23 responsiveness. The other component of the IL23R complex, IL-12Rβ1, was similarly expressed across Th1, Th17 and Th17.1 cells, supporting the presence of a functional IL-23 receptor complex on Th17 and Th17.1 cells.

A key finding of this study is the unexpected increase in IFN-γ secretion by purified human memory CCR6^+^ Th cells and more specifically Th17.1 cells upon IL-23 stimulation. This challenges the traditional view that IL-23 primarily drives IL-17A responses in human CCR6^+^ T cells. This observation supports the notion that IL-23 can promote Th1-like effector functions in Th17.1 cells, a population characterized by co-expression of RORγt and T-bet. Induction of IFN-γ following IL-23 signaling has previously been reported in NK cells (21) and MAIT cells (22), indicating that this effect is not restricted to adaptive immunity. Furthermore, IFN-γ has been shown to induce IL-23 upon stimulation of psoriatic skin, providing a potential local proinflammatory feedback loop (23). Given the central role of IFN-γ in activating innate immune pathways and amplifying inflammatory cascades, its induction by IL-23 in Th17.1 cells may represent an additional mechanism contributing to PsA pathogenicity.

In marked contrast to the response of purified Th17.1 cells, exogenous IL-23 mediated a reduction of IFN-γ secretion by Th17.1 cells in the presence of autologous monocytes. Blocking endogenous IL-23 in these co-cultures had no effect on IFN-γ levels. Simultaneously, IL-23 acted as a potent inducer of IL-17A when Th17.1 or Th17 cells were cultured together with monocytes. This indicates a dose-dependent effect of IL-23 on Th17.1 cells, whereby strong IL-23 signaling can shift these Th17.1 cells toward IL-17A production at the expense of IFN-γ, but that the IL-23 levels required to do this are not obtained in our monocyte-cocultures. Notably, co-culture with monocytes enhanced IFN‑γ production in Th1 and Th17.1 cells despite the absence of detectable IL‑12, indicating that this effect is not driven by canonical IL‑12 signaling. At the same time, IL‑1β and IL‑6 were consistently detected across all co-culture conditions, supporting the presence of a mixed set of monocyte-derived signals that can differentially shape T cell effector functions, including promotion of IL‑17A production. Within this context, IL-23 signaling remained a key driver of IL‑17A production, as its blockade markedly reduced IL-17A secretion in both Th17 and Th17.1 cocultures, providing mechanistic support for the clinical efficacy of IL-23 inhibitors in PsA. (24).

The pathogenic roles of both IFN-γ and IL-17A are underscored by their complementary effects on synovial fibroblasts. IFN-γ induces CD40, CD54, and MHC class II to enable antigen presentation (25), costimulatory signaling, and stable T-cell adhesion, and IL-17A amplifies this inflammatory phenotype by promoting cytokine release and matrix-degrading enzyme production (26). Together these cytokines drive chronic tissue inflammation and remodeling. Both IL-17A and IFN-γ are involved in bone remodeling homeostasis via their effects on osteoclast and osteoblast formation, which is a key symptom of psoriatic arthritis (26). IFN-γ can favor bone remodeling by promoting osteoclast activation (27), while IL-17A can influence both bone formation by inducing osteoblast differentiation, proliferation and mineralization, and bone degradation by stimulating osteoclastogenesis through increased RANKL expression and activation of osteoclast precursors (28). However, at the elevated IL-17A levels typically present in inflammatory arthritis, its effect is predominantly skewed toward bone resorption through enhanced osteoclastogenesis (29). From this perspective, our data suggest that IL-17A^high^IFN-γ^high^ Th17.1 cells are uniquely positioned to couple stromal activation with enhanced bone turnover, thereby reinforcing both inflammatory and structural components of joint pathology in psoriatic arthritis. A limitation of our study is that we assessed the effect of IL-23 stimulation and blockade only in unseparated PBMCs from treatment-naïve PsA patients. This approach does not allow us to distinguish between the specific contributions of Th17.1 cells compared to Th17 or Th1 cells in IL-17A and IFN-γ secretion. Our use of a monocyte co-culture system with cells from healthy controls addresses this limitation, enabling us to study IL-23 within a physiologically relevant context and ensuring that the observed effects reflect complex immune interactions rather than isolated cells. Another limitation is the absence of reliable surface IL-23 receptor staining (2), which would have allowed us to identify IL-23-responsive cells and assess heterogeneity in receptor expression. Despite these shortcomings, the reproducibility of differential IL-23 signaling across our experiments and cell types strongly indicate the stable presence of a functional receptor in both Th17 and Th17.1 cells.

Future work focusing on the effect of IL-23 signaling could include stimulating T helper cells in the presence of antigen-presenting cells and incorporating joint-specific components such as synovial fibroblasts. Such approaches would allow investigation of Th17.1 cells within a microenvironment that closely mimics the *in vivo* conditions, providing deeper insight into their role in chronic inflammation. In addition, further studies are needed to dissect the mechanisms underlying the shift from IL‑23–associated IFN‑γ production toward IL‑17A upregulation in Th17.1 cells. This could be addressed by selectively blocking or adding IL‑6 or IL‑1β, or by using transwell systems to distinguish between soluble factors and cell–cell contact–dependent signals.

Together, this study underscores how IL-23 signaling and monocyte interaction reshape the identity of human Th17.1 cells from an IL-17A^low^ IFN-γ^high^ into an IL-17A^high^ IFN-γ^high^ profile. This context-dependent plasticity highlights the complexity of IL-23 signaling in Th17 biology and suggests that therapeutic strategies targeting IL-23 must consider its dual capacity to amplify both Th17-like and Th1-like programs in autoimmune settings such as PsA.

## Data Availability

The raw data supporting the conclusions of this article will be made available by the authors, without undue reservation.

## Supplementary material

Supplementary Table 1 Baseline characteristics of patients with psoriatic arthritis.

Supplementary Figure 1 Gating strategy for identification and sorting of CD4^+^ T-cell subsets. Representative flow-cytometry gating strategy used to identify CD4+ T-cell subsets. Cells were first gated on lymphocytes based on FSC-A and SSC-A, followed by exclusion of doublets using FSC-A versus FSC-H. Live cells were selected by exclusion of LD-DAPI^+^ events. CD3^+^ T cells were subsequently identified, and CD4^+^CD45RO^+^ memory T cells (CD4 Tmem) were gated. Regulatory T cells were excluded based on CD25 and CD127 expression. CD4^+^ memory non-Treg cells were subdivided according to CCR6 expression. Within the CCR6^+^ population, Th17 (CCR6^+^CXCR3^–^CCR4^+^) and Th17.1 (CCR6^+^CXCR3^+^CCR4^-^) cells were identified, while Th1 cells (CCR6^–^CXCR3^+^CCR4^-^) were defined from the CCR6^–^ fraction. For downstream experiments, either CCR6^+^ and CCR6^–^ populations or Th17, Th1, and Th17.1 subsets were sorted.

Supplementary Figure 2 Intracellular IFN-γ and IL-17A staining on sorted Th17.1 cells following IL-23 stimulation. Sorted human memory Th17.1 cells were stimulated with αCD3/αCD28 antibodies in the presence or absence of IL-23. Cells were subsequently restimulated with PMA, ionomycin, and brefeldin A for 4 hours prior to intracellular cytokine staining. The percentage of IL-17A^+^ or IFN-γ^+^ cells, as well as the mean fluorescence intensity (MFI) of IL-17A and IFN-γ, is shown. Each data point represents an individual donor (n=3).

Supplementary Figure 3 Monocytes in monoculture do not produce IL-17A or IFN-γ. Monocytes were cultured in monoculture. IL-17A and IFN-γ levels were measured in culture supernatants after 72 hours. Data represents three independent experiments, with a total sample size of n = 9.

Supplementary Figure 4 Intracellular IFN-γ and IL-17A staining on sorted Th17.1 cells following IL-23 stimulation in coculture with monocytes. Sorted human memory Th17.1 cells were co-cultured with monocytes and stimulated with or without IL-23. Cells were subsequently restimulated with PMA, ionomycin, and brefeldin A for 4 hours prior to intracellular cytokine staining. The percentage of IL-17A^+^ or IFN-γ^+^ cells, as well as the mean fluorescence intensity (MFI) of IL-17A and IFN-γ, is shown. Each data point represents an individual donor (n=3).

Supplementary Figure 5 IL-12, IL‑1β and IL-6 secretion by monocytes cocultured with sorted Th17, Th17.1, and Th1 cells. Sorted human memory Th17, Th17.1, and Th1 cells were co-cultured with monocytes and stimulated with or without IL-23. IL‑1β, IL-6 and IL-12 levels were measured in culture supernatants after 72 hours. Data represents three independent experiments, with a total sample size of n = 6-9.
